# Battery‐Supercapacitor Hybrid Devices: Recent Progress and Future Prospects

**DOI:** 10.1002/advs.201600539

**Published:** 2017-02-21

**Authors:** Wenhua Zuo, Ruizhi Li, Cheng Zhou, Yuanyuan Li, Jianlong Xia, Jinping Liu

**Affiliations:** ^1^ School of Chemistry Chemical Engineering and Life Science and State Key Laboratory of Advanced Technology for Materials Synthesis and Processing Wuhan University of Technology Wuhan Hubei 430070 P. R. China; ^2^ Institute of Nanoscience and Nanotechnology Department of Physics, Central China Normal University Wuhan Hubei 430079 P. R. China; ^3^ School of Optical and Electronic Information Huazhong University of Science and Technology Wuhan 430074 P. R. China

**Keywords:** battery‐supercapacitor hybrid, energy/power density, future prospects, multifunctional, recent progress

## Abstract

Design and fabrication of electrochemical energy storage systems with both high energy and power densities as well as long cycling life is of great importance. As one of these systems, Battery‐supercapacitor hybrid device (BSH) is typically constructed with a high‐capacity battery‐type electrode and a high‐rate capacitive electrode, which has attracted enormous attention due to its potential applications in future electric vehicles, smart electric grids, and even miniaturized electronic/optoelectronic devices, etc. With proper design, BSH will provide unique advantages such as high performance, cheapness, safety, and environmental friendliness. This review first addresses the fundamental scientific principle, structure, and possible classification of BSHs, and then reviews the recent advances on various existing and emerging BSHs such as Li‐/Na‐ion BSHs, acidic/alkaline BSHs, BSH with redox electrolytes, and BSH with pseudocapacitive electrode, with the focus on materials and electrochemical performances. Furthermore, recent progresses in BSH devices with specific functionalities of flexibility and transparency, etc. will be highlighted. Finally, the future developing trends and directions as well as the challenges will also be discussed; especially, two conceptual BSHs with aqueous high voltage window and integrated 3D electrode/electrolyte architecture will be proposed.

## 1 Introduction

**Figure 1:**
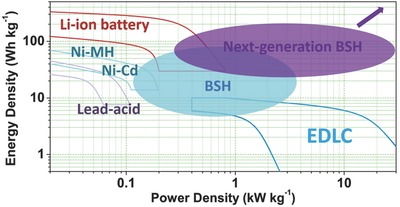
Ragone plots of various rechargeable batteries and EDLC, and the comparison with BSHs.

With the increasing concerns of environmental issues and the depletion of fossil fuels, the emergence of electric vehicles and the generation of renewable wind, wave, and solar power are of great importance to the sustainable development of human society.^1^ Therefore, reliable energy storage systems such as batteries and supercapacitors (SCs) are key elements to enable these energy structure evolutions. In addition to traditional lead–acid, Ni–Cd, Ni–MH, lithium ion batteries (LIBs), and SCs, various advanced batteries such as lithium–air/–sulfur,^2^ sodium/aluminum ion batteries^3^, ^4^ and aqueous metal ion batteries^5^ have been emerging, and great efforts have been devoted to optimize their overall performance for future practical applications.^6^ Building better energy storage devices not only depends on the micro‐/nanostructure design of electrode materials but more crucially relies on the device's configuration engineering.^7^ An energy storage system based on a battery electrode and a supercapacitor electrode called battery‐supercapacitor hybrid (BSH)^8^ offers a promising way to construct device with merits of both secondary batteries and SCs, as shown in **Figure** **1**. This hybridization is indispensable to meet with the demands of both higher energy and power densities for powering future multifunctional electronics, hybrid electric vehicles, and industrial equipment.

To understand the design purpose of such a hybrid device, various kinds of batteries should be first introduced and compared with SCs. The earliest rechargeable battery, lead–acid battery was announced in 1859 by Gaston Planté. With the primary goal of achieving longer cycling life and higher specific capacity, Ni—Cd, Ni–MH batteries with energy density of 30–70 Wh kg^−1^ (Figure 1) had been the most common secondary batteries over a century,^9^ and still hold their position in markets. New types of Ni—Fe alkaline batteries are capable of ultrafast charging enabled by using inorganic–carbon hybrid electrode and could deliver a specific energy density higher than 100 Wh kg^−1^.^10^ During 1970s and early 1980s, the prototype of LIBs, first named “lithium rocking‐chair cells,” was proposed and practically demonstrated;^11^, ^12^ commercial LIB based on LiCoO_2_//graphite was introduced in 1991 by Sony Corporation.^13^, ^14^ Over two decades of development, the energy densities of the‐state‐of‐art LIBs are approaching 200 Wh kg^−1^.^15^ The concept of SCs using carbon electrode in sulfuric acid electrolyte was filed by Becker in 1957,^16^ and the first commercial device appeared in 1978 by NEC (Japan) under the energy company SOHIO's license.^17^ SCs were typically used to complement or even replace batteries in practical fields; those with specific energy densities below 20 Wh kg^−1^ and high specific power density above 10 kW kg^−1^ are commercially available.^18^

Lead–acid, Ni—Cd, Ni–MH, and LIBs store energy based on redox reactions in bulk electrode materials; the electrochemical process is slow and diffusion‐controlled. This enables them with high energy density (30–200 Wh kg^−1^) but relatively low power density (usually within 500 W kg^−1^) and poor cycling stability (500–2000 times). Due to the phase transformations of battery electrodes, the cyclic voltammogram (CV) and galvanostatic charge–discharge curves of batteries are characteristic of strong faradaic redox peaks and flat voltage plateaus, respectively.^19^, ^20^ With surface/near‐surface reaction, the charge transfer of SCs is merely limited at the electrolyte/electrode interface, leading to ultrahigh power density (1–10^3^ kW kg^−1^); and the little destruction of the electrode materials' structure ensures ultralong cycle life (even >10^6^ cycles).^18^ Near rectangular CV and linear potential–time response are essential electrochemical features of SCs.^21^

In spite of their different electrochemical characteristics and behaviors, batteries and SCs share similar configurations, both include anode, cathode, electrolyte, separator, and current collector, making the hybridization feasible. The hybridization enables the direct integration of high energy from batteries and high power and long lifetime from SCs. Note that the BSH device in this review excludes the external hybrid that is physical connection of available SC cells with rechargeable battery cells. Such engineering designs were well described in literatures.^22^, ^23^ Internal parallel hybrid device assembled with each electrode containing both capacitive and battery‐type materials^24^ is also beyond the scope of our discussion. Herein, BSH only refers to internal serial hybrid devices with a battery electrode and an SC electrode. Moreover, unlike previous reviews^8^, ^25^ that only reported LIB‐SC hybrid, we will classify BSH into several types based on the electrolyte components and specific energy storage mechanism.

In recent years, with the rapid developments in intelligent and interactive consumer products, electrochemical energy devices with particular functionalities (such as flexibility, transparency, electrochromism, photodetection, shape memory, and self‐healing, etc.) have been attracting considerable attention in a variety of fields.^26^ For example, smart devices with shape memory ability can be deformed into specific shapes and recovered to the previous states under specific conditions.^27^ If combined with the high energy, high power, safety, environmental friendliness of BSHs, it will be a quite promising energy supply option for future wearable devices.^28^ Thus some emerging multifunctional and flexible BSHs will also be presented and discussed in this Review article.

In brief, this Review article is aiming to summarize the recent advances of BSHs, especially on the development of fundamental scientific principles and concepts. The overall content is organized as follows: First, we specifically review the energy storage mechanism and put forward possible configurations of BSHs; then, we will highlight recent progress on materials, structure and performance of different kinds of BSHs, especially non‐Li‐ion BSHs; After that, the emerging multifunctional BSH devices will be briefly introduced; At last, we will end this review with discussing the current limitations, remaining challenges and prospects of future research for BSHs.

## 2 Design Considerations, Structure, and Classification of BSHs

**Figure 2:**
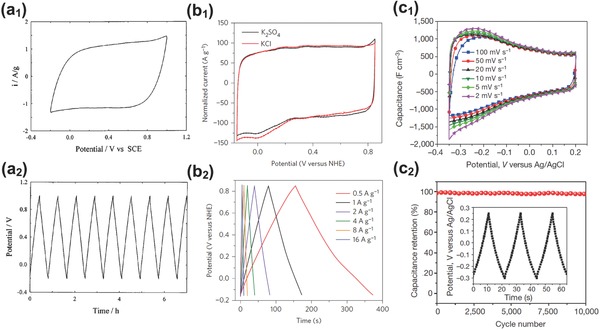
a_1_) CV at 5 mV s^−1^ and a_2_) Charge–discharge curves at 2 mA cm^−2^ for amorphous MnO_2_⋅*n*H_2_O in KCl aqueous electrolyte. Reproduced with permission.^31^ Copyright 1999, Elsevier. b_1_) CV at 10 mV s^−1^ and b_2_) charge–discharge curves for 1T phase MoS_2_ electrode. Reproduced with permission.^40^ Copyright 2015, Nature Publishing Group. c1) CV profiles and c2) cycling stability of Ti_3_C_2_T*_x_* electrode. Reproduced with permission.^41^ Copyright 2014, Nature Publishing Group.

To illustrate the structure details and classification of BSHs and further understand the energy storage mechanism, SCs' electrode is the key and must be distinguished from a battery electrode. Basically, electrode materials of SCs can be classified into electric double‐layer capacitive (EDLC) materials and pseudocapacitive materials. EDLC electrodes store charges via ion accumulation to form electric double layers at the interface between electrode and electrolyte; and the most widely used EDLC materials are various nanocarbons with high specific surface area and relatively low cost.^29^, ^30^ Pseudocapacitive electrodes store energy electrochemically through surface/near‐surface reversible faradaic reactions.^31^ Transition metal compounds (Mn‐/Fe‐/V‐/Mo‐based oxides/hydroxides/sulfides, etc.), conducting polymers (such as polyaniline, polypyrrole, and their derivatives),^32^ and heteroatom (N, O, B, P, etc.) doping carbon‐based electrodes[[qv: 6b]] have been extensively investigated as pseudocapacitive electrode materials. It should be emphasized that pseudocapacitors must possess the basic EDLC‐type electrochemical features.^33^, ^34^ Simon et al. showed their worries about the confusion between battery materials and pseudocapacitive materials and underlined their fundamental electrochemical differences.^19^ In general, qualified pseudocapacitive materials should exhibit a combination of properties that include the followings: (i) strong faradaic reactions at/near surfaces enabling high capacitance; (ii) EDLC‐like electrochemical behaviors over relatively long ranges of potential, that is, near rectangular CV plots and linear potential–time response of charge–discharge curves; (iii) fast charge storage kinetics that offers high power density (>10^3^ W kg^−1^). Nevertheless, there is also “intercalation pseudocapacitance” that occurs via bulk intercalation like batteries but on the same timescale as redox pseudocapacitance, as well elaborated by Dunn and co‐workers^6^, ^35^, ^36^ Conventional RuO_2_ 37, 38 and MnO_2_ 31, 39 and newly emerged materials such as Nb_2_O_5_,^36^ metallic 1T MoS_2_,^40^ LaMnO_3_,^21^ and MXenes^41^ are eligible pseudocapacitive materials exhibiting good capacitive features, as shown in **Figure** **2**. By contrast, many Ni‐ and Co‐based compounds that follow diffusion‐controlled electrochemical process in aqueous alkaline electrolytes and display apparent plateau in charge‐discharge curves cannot be considered as pseudocapacitive materials.

**Figure 3:**
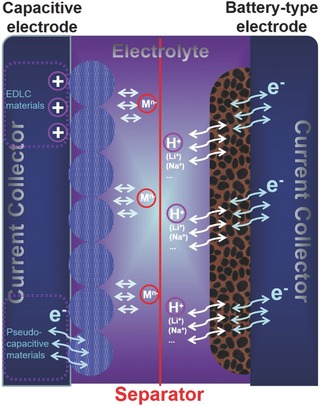
General energy storage mechanism and device structure of BSHs.

**Figure 4:**
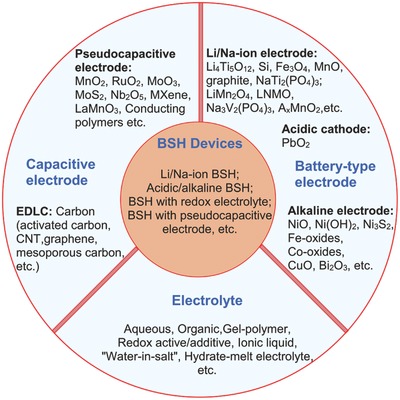
Various types of BSHs and their electrode and electrolyte materials.

With the above consideration, we can expatiate the energy storage mechanism of BSHs as follows: During charging or discharging, anions and cations move to (or separated from) the two electrodes, respectively, and bulk redox reactions occur at the battery‐type electrode while ion accumulation/separation or rapid charge transfer happen at the capacitive electrode; at the meantime, the electrons flow across the external circuit (**Figure** **3**). This design contains one battery electrode and one capacitive electrode, and the capacitive electrode can either be EDLC‐type or pseudocapacitive and can either be anode or cathode. Despite being asymmetric, BSH is different from conventional “asymmetric SCs,” in which both electrodes are capacitive but with asymmetric capacitive charge storage mechanism. There are great opportunities to design various types of BSHs considering the diversity of electrode, electrolyte, and device configuration. Herein, we will classify BSH devices into the following types: Li ion BSH, Na ion BSH, acidic BSH, alkaline BSH, BSH with redox electrolytes, and BSH with pseudocapacitive electrode. The candidate electrode/electrolyte materials are also summarized, as displayed in **Figure** **4**.

**Figure 5:**
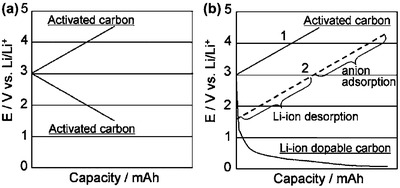
Typical voltage profiles for a) symmetric activated carbon‐based SC and b) activated carbon//Li‐doped carbon BSH. Reproduced with permission.^42^ Copyright 2006, The Electrochemical Society.

In general, BSH devices can surpass the energy density of conventional SCs because of the higher capacity of battery‐type electrode^25^ and could overcome the power density limitation of batteries due to the presence of capacitive electrode as well as the advanced design of battery‐type electrode to ensure fast electrochemical kinetics. The energy density was improved through two approaches: (i) Capacity improvement. Because the capacity of battery‐type electrode is many times higher than that of the capacitive electrode, the capacitance of the BSH devices can usually be improved approximately twofold (or even more) compared with symmetric SCs;^42^, ^43^ (ii) Voltage expansion.^44^ For symmetric SCs, the working voltage of full cell typically could not exceed its electrode's maximum working potential range, that is, the maximum specific capacitance stored in a full cell is only half that of its electrode. By selecting proper battery‐type electrode that works in a separated potential range, full capacitance of the capacitive electrode could be utilized, and the output voltage of the full‐cell BSH device can also be enlarged. For example, as illustrated in **Figure** **5**, a wide potential window of 1.5–4.5 V versus Li/Li^+^ can be used for cathode in a BSH based on intercalation‐type carbon anode and activated carbon cathode; by contrast, only a narrow potential range from 3 to 4.5 V versus Li/Li^+^ has been used for activated carbon cathode in a symmetric SC.^42^ For further information, Choi and Park have given detailed theoretical calculation and comparison of energy and power densities between symmetric SCs and BSHs.^43^

The electrolytes of BSHs could be organic, aqueous, gel‐polymer, or ionic liquid. Organic electrolytes usually include LIB electrolytes (LiPF_6_, LiBF_4_) or sodium ion battery electrolytes (NaClO_4_, NaPF_6_), while aqueous electrolytes could be acidic (H_2_SO_4_, CH_3_SO_3_H), alkaline (KOH, NaOH), and neutral (Li_2_SO_4_, Na_2_SO_4_). Acidic electrolytes are usually utilized for the hybridization of lead–acid battery and SCs; alkaline electrolytes are applied in hybrid devices with one alkaline battery electrode (such as Ni‐/Fe‐/Bi‐oxides/hydroxides) and one nanocarbon‐based capacitive electrode; and neutral electrolytes are mainly employed for aqueous Li‐ion and Na‐ion BSHs. Organic electrolytes have a wide and stable electrochemical voltage window and even allow the operating voltage up to 4.5 V for BSHs (e.g., “activated carbon//graphene” device),^44^ but they are volatile and flammable. Ionic liquids as nonvolatile, highly stable electrolytes can replace the traditional organic ones for BSH applications. Finally, gel–polymer electrolytes can be introduced into BSHs for compact and safe energy storage (minimizing the device's volume without using additional separator and avoiding electrolyte leakage); they are also very popular for designing flexible/stretchable or even smart BSHs.

Considering the electrolyte/electrode types and materials resources, aqueous BSHs (acidic/alkaline BSH, aqueous Li‐ion, and redox BSHs, etc.) might be generally cheaper than organic electrolyte and ionic liquid‐based BSHs. In addition, non‐Li‐ion BSHs such as Na ion BSH with either EDLC or pseudocapacitive electrode will be very promising for long‐term large‐scale applications considering the abundant sodium resources and low cost.

## 3 Recent Advances on Materials and Performance of BSHs

### 3.1 Li‐Ion BSH

LIB is the most widely used electrochemical energy storage device, especially in portable electronics and hybrid electric vehicles.^45^, ^46^ Thus it is hardly surprising that one of the earliest hybrid SCs is Li‐ion based BSH, which was named, reported, and patented by Amatucci et al. in 2001.^47^, ^48^ This Li‐ion BSH was assembled with a nanostructured Li_4_Ti_5_O_12_ anode and an activated carbon cathode. With the high specific capacity of Li‐ion type electrode, Li‐ion BSHs bridged the gap between LIBs and SCs and attracted worldwide attention.

Electrolytes of Li‐ion BSHs can be either organic or aqueous. Benefiting from their high voltage window, theoretically, Li‐ion BSHs based on conventional nonaqueous LIB electrolytes have higher specific energy densities and have aroused enormous research interests. Anode materials such as Ti‐based oxides/compounds (TiO_2_ 49 and Li_4_Ti_5_O_12_,^50^, ^51^ etc.), silicon,^52^ Fe_3_O_4_,^53^ Nb_2_O_5_,^54^, ^55^ MnO,^56^ and intercalated carbon, and cathode materials of LiNi_0.5_Mn_1.5_O_4_,^57^, ^58^ Li_3_V_2_(PO_4_)_3_,^59^ spinel‐LiMn_2_O_4_,^60^ Li_2_CoPO_4_F,^61^ and their hybrids with various conducting carbon/polymers were investigated for Li‐ion BSHs. Among these, one of the most popular commercialized BSHs was a prelithiated carbon anode assembled with an activated carbon cathode, which achieved a specific energy density of 15 Wh kg^−1^ and a cell voltage of 3.8 V.^17^ With almost the lowest lithium intercalation potential and highest cycling stability among all viable Li‐ion anodes, prelithiated carbon materials such as graphite^62^ are one of the most suitable anodes for organic Li‐ion BSHs. Furthermore, through predoping of lithium ions, the activated carbon//prelithiated graphite full cells could avoid electrolyte consumption at the anode side, which was faced by conventional activated carbon//graphite BSHs. For instance, Zhang et al. reported that by using commercial activated carbon as cathode and mesocarbon microbeads as anode, remarkable maximum energy density (92.3 Wh kg^−1^) and power density (5.5 kW kg^−1^) could be achieved,^62^ but in such a BSH device the very low intercalation potential at anode may cause lithium dendrites formation at the electrolyte–anode interface and associated electrolyte decomposition, resulting in cycling decadence and safety hazard. In recent years, companies including Subaru and Fuji heavy industries Ltd, Asahi kasei corporation, etc. have aimed to produce high‐performance predoped Li‐ion BSHs.

Nanostructured hybrid electrodes represent a promising avenue to obtain advanced energy storage devices with excellent performance. With this, enhanced electrochemical properties such as fast charge transfer kinetics and ultralong cycling stability were realized, as presented in previous reviews.^63^, ^64^ The battery‐type electrode's performance for BSH could be enhanced in such a way. For example, hybridized nanostructured electrodes of battery‐type materials with advanced carbon were demonstrated the most effective to achieve ultrafast charging and discharging. Li_4_Ti_5_O_12_‐based Li‐ion BSH is the most widely studied hybrid device since the Ti‐based anodes can completely avoid the electrolyte decomposition (plateau potential >1.5 V vs Li/Li^+^). It reached its milestone in 2013 when Naoi utilized “ultracentrifuging treatment” to attain “nano‐Li_4_Ti_5_O_12_/carbon” anode. This nanoelectrode even showed a high capacity of 78 mAh g^−1^ at 1200 C rate (3 s charge–discharge) and the full cell could reach an energy density of 28 Wh L^−1^ at 10 kW L^−1^,^65^ which shed light on how to design high‐performance battery‐type electrodes for BSHs. A similar concept was also demonstrated for Nb_2_O_5_ anode;^54^, ^55^ with the design of mesoporous or core–shell structured Nb_2_O_5_/carbon anodes, the BSH devices exhibited very high power densities, at which high energy densities were still maintained (e.g., 15 Wh kg^−1^ at 18510 W kg^−1^).

**Figure 6:**
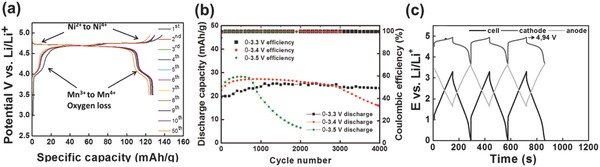
a) Charge–discharge profiles of LiNi_0.5_Mn_1.5_O_4_ cathode in the potential range of 3.50–4.95 V. b) Cycling stability of LiNi_0.5_Mn_1.5_O_4_//AC BSH in different cell voltage ranges. c) Charge–discharge profiles of 3.3 V LiNi_0.5_Mn_1.5_O_4_//AC after 3000 cycles. Reproduced with permission.^58^ Copyright 2014, The Electrochemical Society.

Li‐ion BSHs assembled using battery‐type lithium‐intercalation cathode and capacitive carbon anode also have the advantage of not consuming electrolyte and generally have high output voltage due to the high potential plateau of cathode. Although the specific capacity of intercalation cathode is lower than most of the battery anodes, it still has much higher charge storage ability as compared to the capacitive cathodes in EDLC device and shows great promise to boost the energy density of the BSHs. As is known, the theoretical capacity of layered transition‐metal oxide cathodes are >200 mAh g^−1^; by incorporating Li(Ni_0.5_Co_0.2_Mn_0.3_)O_2_ with single‐wall carbon nanotube by filtration, Wu et al.^66^ obtained a cathode capacity as high as ≈250 mAh g^−1^. With high voltage plateau >4.5 V (**Figure** **6**a), spinel LiNi_0.5_Mn_1.5_O_4_ are promising for BSH devices with higher voltage of ≥3.3 V. Brandt et al. reported a high‐power Li‐ion BSH based on LiNi_0.5_Mn_1.5_O_4_ and activated carbon;^58^ with the cell voltage of 0–3.3 V, this device showed capacity retention of 89% after 4000 cycles (Figure 6b), with average specific energy density and power density of ≈50 Wh kg^−1^ and ≈1100 W kg^−1^, respectively. Figure 6c shows the charge–discharge profiles of LiNi_0.5_Mn_1.5_O_4_//activated carbon device within voltage window of 3.3 V at 600 mA g^−1^ after 3000 cycles. It is clear that the high working voltage of 3.3 V benefited from high potential of intercalation/deintercalation reaction of LiNi_0.5_Mn_1.5_O_4_(≈ 4.8 V vs Li/Li^+^).

Aqueous Li‐ion BSHs is another attractive hybrid device due to the high safety (nonvolatile, nonflammable, and nontoxicity) and high ionic conductivity of aqueous electrolytes as well as the facile device assembly process. They usually use neutral aqueous lithium‐salt solutions (Li_2_SO_4_, LiNO_3_, and LiCl, etc.) as electrolytes. Due to the intrinsic electrochemical stable window of water (≈1.23 V; when the potential is lower than ≈−1.0 V versus SCE, that is, ≈2.3 V versus Li/Li^+^, hydrogen evolution dominates), conventional LIB anode materials such as graphite, TiO_2_, and Li_4_Ti_5_O_12_ are out of choice as BSHs anode except LiTi_2_(PO_4_)_3_.^67^ Nevertheless, lithium transition metal oxide and it analogs^68^ such as LiCoO_2_, LiMn_2_O_4_, LiNi_1/3_Co_1/3_Mn_1/3_O_2_, LiNiPO_4_, etc. are qualified cathode materials for aqueous Li‐ion BSHs. Accordingly, the most popular aqueous Li‐ion BSH configuration is based on lithium transition metal oxide cathode and capacitive carbon anodes. Xia and co‐workers^69^ provided a comprehensive comparison of aqueous Li‐ion BSHs assembled from activated carbon and LiCoO_2_, LiMn_2_O_4_, and LiNi_1/3_Co_1/3_Mn_1/3_O_2_, the effects of pH value on the stability and specific capacity was systematically investigated. But due to the pristine nature of these intercalation cathodes, the reported devices still suffered from slow energy storage kinetics and power densities was constrained within 900 W kg^−1^; the energy density was also limited within 40 Wh kg^−1^.^69^ Nanostructuring and hybridization as aforementioned would be adoptable to promote the overall performance.

### 3.2 Na‐Ion BSH

LIBs have dominated the major electrochemical energy storage markets on portable electronics since 1990s, but lithium is an element whose distribution is far from abundant. Due to the infinite sodium resources and similar physiochemical properties between lithium and sodium, sodium ion energy storage technologies are ideal alternative to LIBs.^70^, ^71^ Sodium ion electrochemical energy storage is not a nascent science and technology, the earliest reversible sodium intercalation material (TiS_2_) was reported in 1980.^72^ In recent years, a large number of sodium‐ion battery electrode materials were demonstrated, such as O3‐layered metal oxide cathode,^73^ Na_3_V_2_(PO_4_)_3_,^74^ Na_2_Fe_2_(SO_4_)_3_,^75^ and Li_4_Ti_5_O_12_,^76^ etc. But unlike LIB electrode materials which usually display fine plateau for charging and discharging, many other sodium ion electrode materials even layered sodium transition metal oxides and their analogues (NaMnO_2_,^77^ Na_0.85_Li_0.17_Ni_0.21_Mn_0.64_O_2_,^78^ Na_x_[Fe_1/2_Mn_1/2_]O_2_,^79^ and Na_0.44_MnO_2_,^80^ etc.) exhibit capacitor‐like features with only sloping plateaus in the charging/discharging curves.

**Figure 7:**
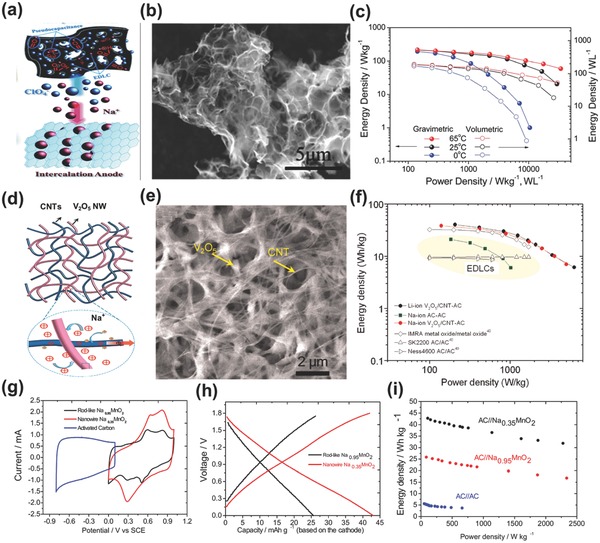
a) Schematic illustration of a carbon//carbon Na‐ion BSH. b) SEM image of the porous nanosheet carbon. c) Ragone plots of the Na‐ion BSH at different working temperatures. Reproduced with permission.^82^ Copyright 2014, The Royal Society of Chemistry. d) Schematic and e) SEM images of V_2_O_5_/CNT nanocomposite. f) Ragone plots of V_2_O_5_/CNT‐based BSH and other devices. Reproduced with permission.^83^ Copyright 2012, American Chemical Society. g) CV curves of AC anode and Na*_x_*MnO_2_ cathode. h) Charge–discharge profiles and i) Ragone plots of Na*_x_*MnO_2_//AC BSH. Reproduced with permission.^84^ Copyright 2013, Elsevier.

Sharing the merits of both Li‐ion BSHs and sodium ion batteries, Na‐ion BSHs have made obvious progress in the last four years. The device configuration can directly refer to Li‐ion BSHs with organic or aqueous electrolytes. Accordingly, carbon//carbon matching is the most widely investigated Na‐ion BSH structure, in which intercalation carbon was used as the anode while capacitive carbon as the cathode. For example, Kuratani et al. reported an Na ion BSH based on Na predoped hard carbon and activated carbon.^81^ However, due to the relatively low capacity of hard carbon in Na‐ion electrolyte, the electrochemical performance of the device is inferior to that obtained in Li‐ion electrolyte. In order to improve the device's performance, the delicate design of micro‐/nanostructures of electrodes is very important. In general, fast Na intercalation kinetics and high surface area should be ensured for battery‐type carbon anode and capacitive carbon cathode, respectively. With both electrodes derived from peanut shells (a green waste generated with large amounts), Ding et al.^82^ assembled a carbon//carbon Na‐ion BSH, in which the resulting carbon materials are highly flaky, thin and porous (**Figure** **7**a,b). Specifically, anode carbon was designed with partially ordered thin graphene domains while cathode carbon was with large surface area and high levels of oxygen doping, providing fast Na^+^ diffusion and abundant redox active sites as well as massive ion accumulation and pseudocapacitive reaction. As a result, very low potential hysteresis between charge and discharge processes was attained for anode and ideal EDLC behavior of the cathode was achieved. Finally, this Na‐ion BSH exhibited excellent energy and power densities (201, 76, and 50 Wh kg^−1^ at 285, 6500, and 16 500 W kg^−1^, respectively), as shown in Figure 7c. Even working within a wide temperature range (0–65 °C), the device still demonstrated attractive energy and power combinations.

Several other sodium ion battery electrodes were used as battery‐type electrode for Na‐ion BSHs, such as V_2_O_5_,^83^ sodium titanate,^85^ Na*_x_*MnO_2_,^84^, ^86^ K*_x_*MnO_2_,^87^ cobalt hexacynoferrate (CoHCF),^88^ Na_4_Mn_9_O_18_,^89^ etc. Among them, α‐V_2_O_5_ could accommodate a variety of metal ions (Li^+^, Na^+^, K^+^, etc.) due to its layered structure. However, the poor electrical conductivity hindered its energy storage applications. To address this issue, Chen et al.^83^ constructed a porous composite architecture which consists of interpenetrating networks of V_2_O_5_ nanowires and carbon nanotubes (CNTs), as shown in Figure 7d,e. Benefiting from the effective electron transport enabled by highly conductive CNT network and fast pseudocapacitive charge storage process due to small dimension of V_2_O_5_ nanowire, the assembled Na‐ion AC//V_2_O_5_‐CNT device delivered an energy density of 38 Wh kg^−1^ at power density of 140 W kg^−1^ (Figure 7f). The overall performance was even comparable to that of the Li‐ion AC//V_2_O_5_‐CNT device. In another example, Wu and co‐workers^84^ investigated the electrochemical application of Na_x_MnO_2_ in Na‐ion BSH, and found that nanowire Na_0.35_MnO_2_ had better Na‐insertion peaks and higher capacity than rod‐like Na_0.95_MnO_2_ (Figure 7g). The Na_0.95_MnO_2_//AC device exhibited 1.8 V output voltage, well‐defined capacitive behavior, and a high energy density of 42.6 Wh kg^−1^(Figure 7h,i).

**Figure 8:**
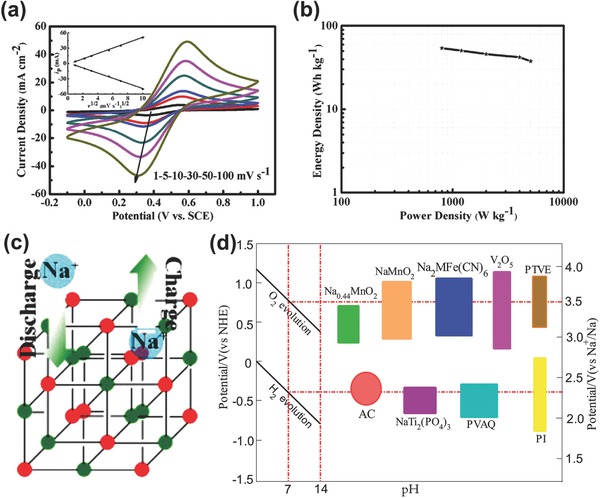
a) CV curves of CoHCF. b) Ragone plots of CoHCF//CMS Na‐ion BSH. c) Wide channels in CoHCF allow for rapid insertion/removal of Na^+^. Reproduced with permission.^88^ Copyright 2015, Elsevier. d) Typical anodes and cathodes for aqueous Na‐ion BSHs.^91^

Recently, a novel aqueous Na‐ion BSH constructed from carbon microspheres (CMSs) and CoHCF was reported.^88^ As in **Figure** **8**a, the strong redox CV peaks at various scan rates indicate that in Na_2_SO_4_ electrolyte CoHCF exhibited distinct battery‐type feature with fast electrochemical kinetics. The Ragone plot of CoHCF//CMS BSH is shown in Figure 8b, ultrahigh energy and power densities (such as 37.8 Wh kg^−1^ at 5037 W kg^−1^) were achieved. CoHCF belongs to Prussian Blue analogues (PBAs). Its crystal structure consists of a face‐centered cubic framework of transition metal cations octahedrally coordinated to hexacyanometallate groups. The above good electrochemical performance was attributed to the wide channels in CoHCF that allow for the rapid insertion/removal of alkali cations with little lattice strain (Figure 8c). With aqueous electrolytes, only limited electrode materials can be utilized in Na‐ion batteries; thus, there are not so many battery‐type electrode materials for aqueous Na‐ion BSHs. Figure 8d summarizes the typical available anodes (NaTi_2_(PO_4_)_3_, poly(2‐vinylanthraquinone): PVAQ, Polyimide: PI, etc.) and cathodes (Na*_x_*MnO_2_, PBAs, V_2_O_5_, etc.)^90^, ^91^ that could be considered for designing future aqueous Na‐ion BSHs.

Similar to the design of Li‐ion BSH and Na‐ion BSH, it is also possible to fabricate high‐performance K‐ion BSH device. Although various capacitive electrodes from Li‐ and Na‐ion BSHs may be utilized, there are still huge challenges from the K‐ion intercalation battery‐type electrodes, as the ionic radius of K^+^ is much larger than that of Li^+^/Na^+^ and thus the host materials are very lacking. Nevertheless, some recent works on advanced K‐ion battery electrodes of K*_x_*MnO_2_,^89^ hexacyanoferrates,[[qv: 4b]] and K_2_C_6_O_6_[[qv: 4c]] have shed great light on designing K‐ion BSH.

### 3.3 Acidic BSH

As one of the three most important commercial rechargeable batteries, lead–acid battery was developed over one and a half century and still occupies its position in energy storage research and application. Patents and papers of lead–acid batteries still emerge in recent years. The hybridization of lead–acid battery with SC leads to acidic BSH. The first acidic BSH (also referred to as “lead–carbon capacitor”) was patented in 2001^92^ and is now commercially available. Lead–carbon capacitor was the only hybrid system based on strong aqueous acidic electrolytes, which utilized a mixture of lead dioxide and lead sulfate as positive electrode and activated carbon as negative electrode.^93^ Among various BSHs, lead–carbon capacitor is superior regarding its high voltage (≈2.0 V); furthermore, recycling PbO_2_ and sulfuric acid is now well mastered and financially self‐sufficient. The charging–discharging process of positive electrode (PbO_2_) is based on the redox couple of Pb^2+^/Pb^4+^, while high‐surface‐area carbon anode stores charge by ion adsorption/desorption. Benefiting from the well‐established battery technologies, the lead–carbon capacitor has advantages of low price and long cycling stability over 10 000 cycles.^22^, ^45^ Nevertheless, like lead–acid battery, lead–carbon capacitor suffers from low specific energy density (15–30 Wh kg^−1^) and low power density due to the limited electrochemically active surface of PbO_2_ film,^94^, ^95^ which hinders the rapid redox reactions. Recent attempts have been made to optimize the performance. For instance, Guay and co‐workers^96^ designed a 3D nanowire arrayed PbO_2_, which enabled a very high charging rate of 22 C; at this rate, the capacitor was still cycled over 5000 times. Gao and co‐workers reported the use of graphite substrate to support PbO_2_ film growth and found that the graphite current collector improved the electrode stability in an H_2_SO_4_ electrolyte; the device could suffer from ≈3000 deep cycles at 10 C with an energy density of 27 Wh kg^−1^.^95^

### 3.4 Alkaline BSH

Nickel–cadmium batteries (Ni–Cd) were invented alongside lead–acid battery and have been used for ≈100 years; it is the prototype and the most mature system of alkaline batteries. In recent years, the emerging Ni, Co, and Fe‐based alkaline batteries with fine charge–discharge voltage plateaus have also been attracting great interests. Alkaline BSHs refer to the hybrid systems that employ alkaline aqueous solutions as electrolytes and carbon materials as the capacitive electrode; the other electrode is based on transition metal oxides/hydroxides (even sulfide, nitride, phosphide) which exhibit battery‐like performance. In general, the capacity of transition metal compounds in basic electrolytes can reach 200–400 mAh g^−1^, typically higher than aqueous Li‐ or Na‐ion battery electrodes. As a result, alkaline BSHs were developed to pursue high energy densities.

**Figure 9:**
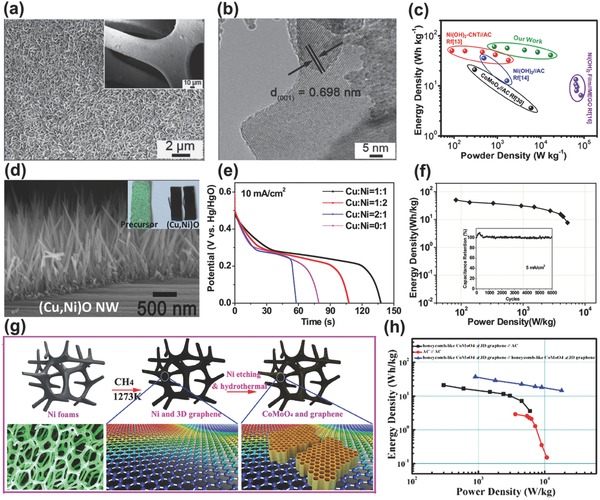
a) SEM and b) HRTEM images of NiMoO_4_ nanosheets (NMO‐NS). c) Ragone plots of NMO‐NS//AC alkaline BSH device. Reproduced with permission.^103^ Copyright 2015, Wiley‐VCH. d) SEM image of (Cu,Ni)O nanowire array. e) Discharge curves and f) Ragone plot/cycling performance of (Cu,Ni)O//AC alkaline BSH. Reproduced with permission.^106^ Copyright 2016, The Royal Society of Chemistry. g) Schematic illustration of the synthesis process of CoMoO_4_‐3D graphene hybrid electrode. h) Ragone plot of CoMoO_4_//AC BSH. Reproduced with permission.^107^] Copyright 2014, Wiley‐VCH.

The first patent of alkaline BSH is two years earlier than Amatucci's Li‐ion BSH.^48^, ^97^ Although with terminology improper, Stepanov et al. provided the earliest nickel oxide//carbon device in potassium hydroxide electrolyte.^98^ Until now, with the redox potential within 0.1–0.6 V (vs SCE), Ni‐based compounds such as NiO,^99^, ^100^ Ni(OH)_2_,^101^, ^102^ NiMoO_4_,^103^ NiCoO_2_,^104^ Ni_3_S_2_,^105^ and their hybrids with conductive carbon or polymers are still the most popular cathode materials for aqueous alkaline BSHs. Beneficial from the fast development of nanotechnology, the comprehensive electrochemical properties have been greatly improved with respect to the first alkaline BSH (≈15 Wh kg^−1^). Kolathodi et al.^99^ reported an alkaline BSH device based on NiO nanofibers and activated carbon, which delivered a high energy density of 43.75 Wh kg^−1^ and demonstrated long cycling life of 5000 cycles. Another device reported by Yan et al.^101^ using Ni(OH)_2_/graphene cathode and porous graphene anode exhibited ultrahigh energy density of 77.8 Wh kg^−1^ at 174.7 W kg^−1^. Lou and co‐workers^103^ developed ternary NiMoO_4_ nanosheet/nanowire array electrodes (**Figure** **9**a,b) at different substrates. The resulting NiMoO_4_//activated carbon alkaline BSH delivered quite high energy and power densities (60.9 Wh kg^−1^ at 850 W kg^−1^ and 41.1 Wh kg^−1^ at 17002 W kg^−1^), as shown in Figure 9c.

Taking the advantages of nanowire array and the synergistic interaction between NiO and CuO at the nanoscale, the unique integrated copper–nickel oxide ((Cu,Ni)O) mesoporous nanowire array cathode (Figure 9d) was designed and fabricated by our group.^106^ This hybrid electrode's electrical conductivity and electroactivity can be readily tuned with the Cu: Ni ratio (Figure 9e), and the optimized electrode achieved a high capacity of ≈260 mAh g^−1^. Furthermore, by coupling with AC anode, the established (Cu,Ni)O//AC alkaline BSH device exhibited outstanding performance in terms of high energy density (50.3 Wh kg^−1^), high power density (5313.4 W kg^−1^) and excellent cycling stability (100.9% capacity retention after 6000 cycles), as demonstrated in Figure 9f and its inset. Beside Ni‐based materials, Co‐based compounds have similar plateau potentials and were also extensively investigated as cathode for alkaline BSH.^108^, ^109^ For example, Yu et al. prepared a novel nanohoneycomb‐like CoMoO_4_‐3D graphene nanostructure cathode by chemical vapor deposition and subsequent hydrothermal reaction (Figure 9g).^107^ With stable structure, high electrical conductivity, and large surface area, this electrode displayed potential plateau within 0.1–0.4 V and could even be charged at very high current density of 85.71 A g^−1^. The resulting CoMoO_4_‐3D graphene//activated carbon device displayed maximum energy density of 21.1 Wh kg^−1^ and power density of >10^4^ W kg^−1^ as well as outstanding cyclability (87.4% after 10 000 times, Figure 9h).

**Figure 10:**
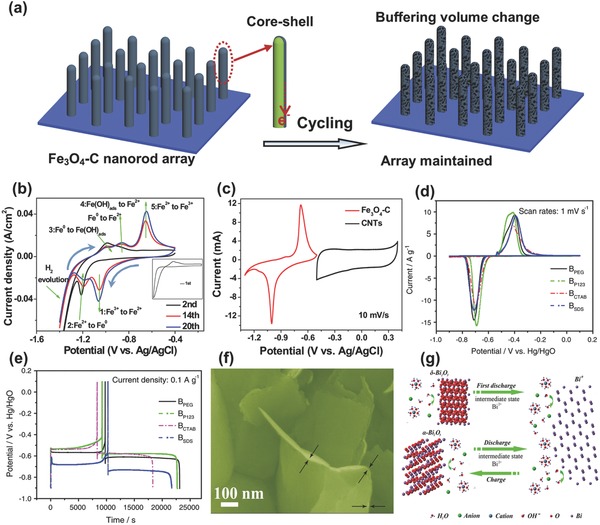
a) Schematic illustration of Fe_3_O_4_—C electrode's merits. b) Energy storage mechanism of Fe_3_O_4_—C electrode in KOH electrolyte. c) Comparative CV curves of Fe_3_O_4_—C anode and CNTs cathode in three‐electrode cells. Reproduced with permission.^116^ Copyright 2015, Wiley‐VCH. d) Typical CV and e) charge–discharge curves of Bi_2_O_3_ electrode in alkaline electrolyte. Reproduced with permission.^117^ Copyright 2014, Springer. f) SEM image of Bi_2_O_3_ nanosheet film. g) Energy storage mechanism of Bi_2_O_3_ in aqueous electrolytes. Reproduced with permission.^5^ Copyright 2016, The Royal Society of Chemistry.

Iron oxides are one of the few anode candidates for rechargeable alkaline battery (such as Ni–Fe batteries) due to its low cost, environmental friendliness and natural abundance. However, the application of iron oxides in alkaline BSHs was rarely investigated. The shortcomings of Fe‐oxide anodes are their relatively low conductivity and pulverization with cycling or at high current densities (huge volume expansion). Various strategies were applied to address these issues, such as core/shell structure design,^110^, ^111^ and hybridization with nanocarbon/conducting polymer, etc.^112^, ^113^, ^114^, ^115^ Recently, to establish an Fe‐based high‐performance alkaline BSH device, our group^116^ put forward a “carbon shell‐protection” solution and synthesized an Fe_3_O_4_–carbon binder‐free nanorod array anode. With facile carbon coating, the volume expansion and the possible structural deformation were effectively restricted, helping maintaining the electrode's integration; the carbon shell also improved the electrical conductivity (**Figure** **10**a). To understand the electrode electrochemistry, the component evolution at different CV stages and the energy storage mechanism of the Fe_3_O_4_–carbon anode in KOH electrolyte were systematically investigated. A “Fe^3+^ ⇌ Fe^0^” conversion process during charging/discharging was unveiled, as illustrated in Figure 10b. This hybrid anode worked within suitable potential range, which matched well with that of capacitive CNTs cathode if comparing the CV profiles (Figure 10c). Consequently, a novel CNTs//Fe_3_O_4_–carbon alkaline BSH with a ≈1.6 V voltage was assembled.^116^

Another promising anode material for alkaline BSHs is bismuth oxide (Bi_2_O_3_). Although used as supercapacitor electrode as early as 2006,^118^ Bi_2_O_3_ received far less attention compared to other alkaline battery electrode materials.^119^ Bi_2_O_3_ typically exhibits superior battery‐type characteristics than Fe‐based oxides; it has stronger CV redox peaks and more even charge–discharge plateaus in basic electrolytes (Figure 10d,e).^117^ In particular, the redox potentials of Bi_2_O_3_ are slightly higher than that of Fe‐oxides; in this regard, the water electrolysis can be avoided more efficiently. Qu et al.^120^ reported a hybrid device constructed using activated carbon as the positive electrode and a “carbon‐nanoparticle Bi_2_O_3_” as the negative electrode in KOH electrolyte. It was found that the capacitance of the device was increased by about 84% in comparison with that of a typical symmetric system. Selvan and co‐workers^121^ used a flower‐like Bi_2_O_3_ as the anode and activated carbon as cathode to construct an alkaline BSH, which demonstrated an energy density of 10.2 Wh kg^−1^ at 1.5 mA cm^−2^. Despite some progress, the working mechanism of Bi_2_O_3_ anodes was not well understood and the overall performance of the device needed significant improvement. Very recently, our group fabricated a film electrode of Bi_2_O_3_ ultrathin nanosheets (binder‐ and additive‐free; Figure 10f), which interestingly exhibited excellent electrochemical characteristics even in over seventeen neutral/near‐neutral aqueous electrolytes with a remarkably high capacity of >360 mAh g^−1^.^5^ A unique “quasi‐conversion reaction” energy storage mechanism that differs from conventional intercalation‐type mechanism was unveiled (Bi_2_O_3_ ↔ Bi^0^) (Figure 10g). This work revealed the great potential of Bi_2_O_3_ in aqueous electrochemical energy storage. It is believed that further designs of the micro/nanostructures of Bi_2_O_3_ electrode (especially to boost the cycling stability) will be favorable to establish outstanding alkaline BSHs.

### 3.5 BSH with Redox Electrolytes

BSHs with redox electrolytes are electrochemical devices that have some similarity to redox‐flow batteries, in which the redox materials are active electrolytes or redox additives dispersed in electrolytes and the redox reactions take place at the electrode–electrolyte interfaces.^122^ The major difference is that in redox‐flow batteries, the redox materials exist in a flowing form while in redox electrolyte BSHs, the electrolytes are relatively static. BSHs with redox active electrolytes are devices whose solvents are used both as redox and conducting mediator; while for BSHs with redox additive electrolytes, electrolytes contain both redox mediator and supporting electrolytes. These kinds of electrochemical energy storage devices could not be called redox‐active/additive SCs because the redox electrolytes exhibit battery‐type features.^123^ Typically, redox electrolyte BSHs employ one kind of redox electrolyte, with which redox reactions occur at one electrode–electrolyte interface (as the battery‐type side); the other electrode is high‐surface‐area carbon that stores charges in a capacitive way. If there are electrolyte redox reactions around both electrodes, the device would evolve into redox batteries.

**Figure 11:**
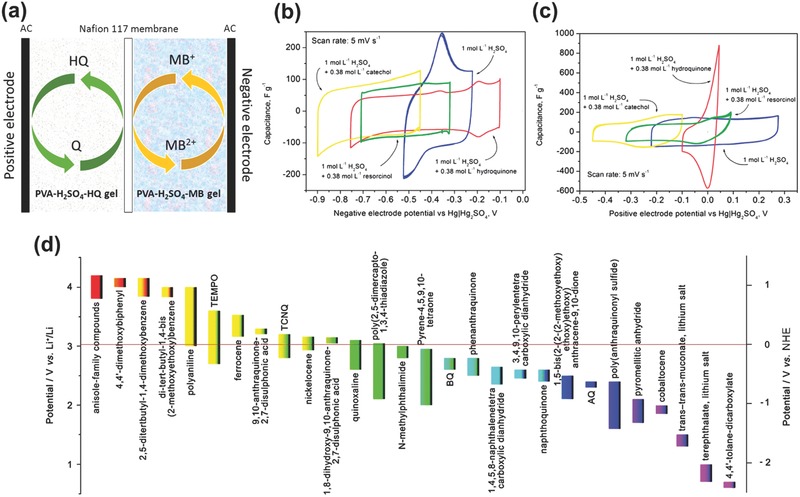
a) Schematic illustration of redox‐additive BSH device of PVA‐H_2_SO_4_‐HQ//PVA‐H_2_SO_4_‐MB. Reproduced with permission.^131^ Copyright 2015, Elsevier. CVs for b) negative electrode and c) positive electrode in dihydroxybenzene‐modified acidic electrolytes. Reproduced with permission.^132^ Copyright 2015, The Royal Society of Chemistry. d) Redox potential of various organic and organometallic candidates. Reproduced with permission.^129^ Copyright 2015, The Royal Society of Chemistry.

**Figure 12:**
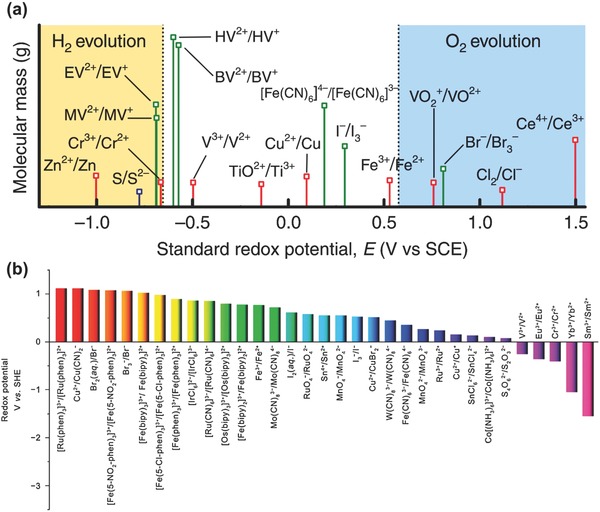
Redox potential of various reversible inorganic redox couples. a) Reproduced with permission.^130^ Copyright 2015, Nature Publishing Group. b) Reproduced with permission.^129^ Copyright 2015, The Royal Society of Chemistry.

Redox electrolyte BSH was first reported in 2010^124^ and aimed to improve the specific capacity and energy density of SCs by integrating the faradaic contribution of the redox electrolyte. The most important principle for designing such a device should be the selection of redox electrolytes. For this, the following demands must be fulfilled: (i) the potential of redox couple must be either highly positive or highly negative to obtain a maximum cell voltage; (ii) the redox reactions should be highly reversible; (iii) the electrolyte should be highly stable, either in oxidized or reduced states, and it should not react with solvent; (d) the solubility of redox‐additive electrolyte in solvent should be high; (e) both active electrolyte and solvent should be cheap, safe, environmental friendly, and abundant. With five to six years of development, various redox electrolytes such as organic small molecules (e.g., quinine/hydroquinone: HQ, methylene blue: MB; **Figure** **11**),^125^, ^126^ inorganic (e.g., KI, Fe(CN)_6_^3−^/Fe(CN)_6_^4−^; **Figure** **12**),^127^, ^128^, ^129^, ^130^ and gel–polymers^131^ were investigated. For example, a quasi‐solid‐state sandwiched energy storage device^131^ based on electrolytes of PVA‐H_2_SO_4_‐HQ gel, PVA‐H_2_SO_4_‐MB gel, and Nafion 117 membrane is displayed in Figure 11a. There are also many new redox species uncovering. Fic and co‐workers examined the electrochemical behavior of different redox active solutions especially dihydroxybenzenes with varying —OH substitutions.^132^ The results showed that different potential range and redox behavior would be achieved by tuning the stereochemistry, i.e., —OH substitution (Figure 11b,c). Similar to other kinds of BSHs, device with organic redox electrolytes holds wider voltage window. The redox potentials of various organic and organimetallic candidates^129^ for redox electrolyte BSHs are shown in Figure 11d, as compared to those of potential inorganic redox couples (Figure 12).

With the contribution from redox active/additive electrolytes, the redox electrolyte BSHs have demonstrated much higher energy density (≈15–45 Wh kg^−1^) than symmetric carbon SCs (≈3–10 Wh kg^−1^). For instance, a maximum energy density of 45 Wh kg^−1^ has been attained for device with Cu^2+^ added [EMIm]BF_4_ electrolyte.^124^ In another example, the addition of decamethylferrocene in electrolyte resulted in 27‐fold increase in energy density of CNT‐based SCs (from 1.3 to 36.8 Wh kg^−1^).^133^ In general, these energy densities are comparable to aqueous Li‐ion BSHs but lower than alkaline BSHs and organic Li‐ion BSHs. Redox electrolyte BSHs still have some drawbacks. Like aqueous redox‐flow batteries, first, the supporting aqueous electrolytes are usually highly corrosive, such as strong acid (H_2_SO_4_) and strong alkali (KOH), and therefore chemically inert electrode materials especially carbon‐based ones are needed.^122^, ^132^, ^134^, ^135^ Second, in order to separate redox electrolyte from the other electrode to avoid self‐discharge, semipermeable membranes must be necessary to separate the cell into two compartments. Third, considering that the electrolytes are active in electrochemical process and the volume or mass of electrolytes play a vital role on the performance of full devices, the calculation of the capacity and energy density should be based on the full device including electrolytes; in this regard, the real energy/power densities will be lower than reported values.

Unlike aqueous and organic electrolytes, ionic liquids offer unparalleled merits such as high safety and wide electrochemical potential window as high as 5.3 V.^136^ As a result, BSH devices based on pure ionic liquids has extended the operating voltage and increased the capacity and energy density.^137^ Furthermore, by adding redox additive electrolytes, the device's performance could be comprehensively improved. As an example, Navalpotro et al.^123^ reported a BSH based on quinone in PYR_14_TFSI, the operating voltage could easily reach 3–3.5 V, and the energy density was increased 300% than using pure PYR_14_TFSI. Room‐temperature ionic liquids provide a great opportunity to construct high‐energy BSHs, but there are also two drawbacks: First, ionic liquids are typically unstable below 1 V (vs Li/Li^+^), which conversely constrains the voltage of full device within ≈4.3 V. Second, the high viscosity reduces ionic conductivity that may result in relatively low power density.

Redox electrolyte BSH is actually a nascent system, although attracting increasing attention, prominent researches are quiet rare. With developing highly qualified redox electrolytes, it is expected that more and more work will be reported on the overall device performance improvement in the near future.

### 3.6 BSH Based on Pseudocapacitive Electrode

**Figure 13:**
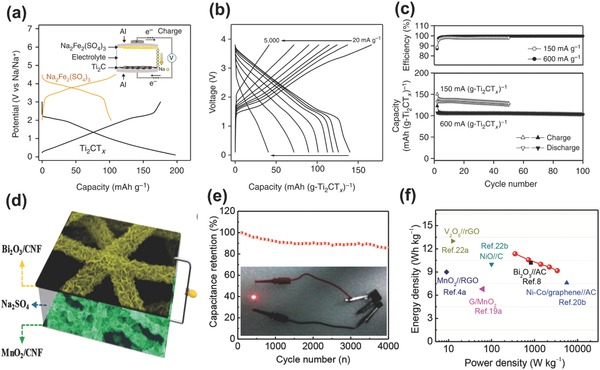
Na_2_Fe_2_(SO_4_)_3_//Ti_2_CT*_x_* BSH device. a) Charge–discharge curves of electrodes, b) charge–discharge plots at various rates, and c) cycling performance and coulombic efficiency for the full cell. Reproduced with permission.^140^ Copyright 2015, Nature Publishing Group. MnO_2_//Bi_2_O_3_ BSH device. d) Schematic illustration, e) cycling performance, and f) Ragone plot. Reproduced with permission.^141^ Copyright 2015, Wiley‐VCH.

In most of BSH devices, carbon‐based materials were utilized as the capacitive electrode; the charges were stored mainly based on an electric‐double‐layer model. Recently, another concept has been developed, which employed pseudocapacitive materials as the capacitive electrode rather than carbon to further improve the energy storage capability of full‐cell devices. Since there are several pseudocapacitive materials such as MXenes,^138^ MoS_2_ 139 that has ultrafast energy storage kinetics comparable to EDLC materials, the hybrid devices based on pseudocapacitive electrodes and battery‐type electrodes could achieve much superior energy density than carbon‐based BSHs while maintaining high power density. A typical example was demonstrated by Wang et al.,^140^ who constructed an Na‐ion BSH device with an organic NaPF_6_/ethylene carbonate‐diethyl carbonate(EC‐DEC) electrolyte based on Ti_2_CT*_x_* MXene nanosheet anode and alluaudite Na_2_Fe_2_(SO_4_)_3_ positive electrode (**Figure** **13**a). In such a design, the pseudocapacitance of the MXene Ti_2_CT*_x_* nanosheet anode achieved a higher specific capacity relative to double‐layer capacitive electrodes (low capacitance) and a higher rate capability relative to ion intercalation bulk electrodes (slow kinetics). The pseudocapacitance of Ti_2_CT*_x_* comes from the fast Na^+^ intercalation/deintercalation into the 2D interlayer space as well as reversible Na^+^ adsorption/desorption onto the surface of each layer/sheet.^140^ As displayed in Figure 13b, when the current density was increased 250 times from 20 to 5000 mA g^−1^, the device could still keep one third of the initial capacity. The device delivered an ultrahigh specific energy density of 260 Wh kg^−1^ at power density of 1.4 kW kg^−1^ (based on the mass of Ti_2_CT*_x_*). In addition, ≈96% of the device's capacity was retained with high coulombic efficiency of ≈99.7% when cycled 100 times at 600 mA g^−1^ (Figure 13c). Huang and co‐workers^141^ also developed a BSH device based on pseudocapacitive MnO_2_/carbon nanofiber(CNF) cathode, battery‐type Bi_2_O_3_/CNF anode and an aqueous Na_2_SO_4_ electrolyte (Figure 13d). Enabled by CNF in both electrodes (high electrical and mechanical adhesion with grown metal oxides), the MnO_2_/CNF//Bi_2_O_3_/CNF device demonstrated 85% capacitance retention after 4000 cycles; the energy density was much higher than many reported capacitive carbon‐based BSHs such as Bi_2_O_3_//AC, NiO//C, and MnO_2_//reduced graphene oxide(RGO), etc. (Figure 13e,f).

As mentioned earlier, eligible pseudocapacitive materials should fulfill the following key requirements. First of all, strong faradaic reaction on the surface/near‐surface to provide high capacitance; then, capacitive behaviors with fast charge–discharge rate capability.^6^ With this respect, 1T MoS_2_,^40^, ^139^ Nb_2_O_5_,^36^ MoO_3_,^35^ MnO_2_,^31^, ^142^ RuO_2_,^143^, ^144^ LaMnO_3_,^21^ and a variety of MXenes,^41^, ^145^ etc. are highly qualified with expected higher capacitance. Moreover, conductive polymers such as polyaniline (PANI), polypyrrole (PPy), polythiophene, and polyindole show great promise for pseudocapacitive electrodes because of their high capacitance.^32^ Despite this, the current BSH devices still mostly use double‐layer capacitive carbon‐based electrode. Even though the capacitance of carbon electrodes has been improved two‐ to threefold (100–500 F g^−1^ in aqueous electrolytes and 100–200 F g^−1^ in organic electrolytes)^146^, ^147^ as compared to decades ago, it is still generally lower than pseudocapacitive materials. It is envisaged that the integration of pseudocapacitive electrode will be a highly promising approach to furthering the development of BSHs.

## 4 Designing Multifunctional BSHs for Future Applications

With the rapid development of portable electronic devices, smart products, artificial intelligence, and micro‐/nanosystems, multifunctional electrochemical energy storage devices with flexible/stretchable/foldable/wearable, transparent, and intelligent functionalities are highly demanded.^26^ The integration of active materials on unusual current collectors/substrates enables electrodes and devices with new physical or chemical functionalities that cannot be achieved with common current collectors/substrates. Among various functional energy storage devices, flexible, and transparent ones are particularly attracting in the near future due to their potential use in smart wearable electronics and optoelectronic systems.

**Figure 14:**
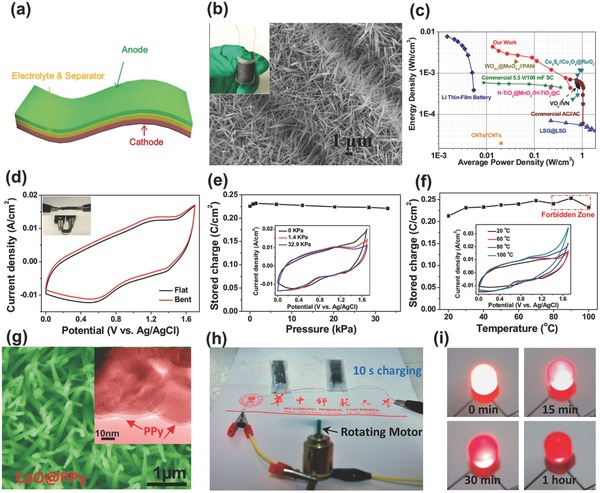
Flexible BSHs. a) Typical configuration of flexible BSH. b) SEM image of Li_4_Ti_5_O_12_ nanowire array anode, the inset is digital photograph of the flexible Li_4_Ti_5_O_12_//CNT BSH. c) Ragone plot of the device. Reproduced with permission.^50^ Copyright 2015, Nature Publishing Group. d–f) Electrochemical stability of CNTs//Fe_3_O_4_‐C BSH device under bending, high mechanical pressure and elevated temperature. Reproduced with permission.^116^ Copyright 2015, Wiley‐VCH. g) SEM image of CoO@PPy nanowire array cathode. h,i) The CoO@PPy//AC BSH powering rotation motor robustly and lighting LED indicator. Reproduced with permission.^152^ Copyright 2013, American Chemical Society.

Flexible electrochemical energy storage devices were designed either directly using flexible free‐standing active material films‐based electrodes such as CNT,^148^ graphene,^149^, ^150^ and nanocarbon‐metallic textile or growing active materials on flexible substrates.[[qv: 26b,d,28,151]] **Figure** **14**a illustrates a typical configuration of electrochemical energy storage devices based on flexible electrodes. In general, either liquid or nonliquid electrolytes can be used, but solid‐state or quasi‐solid‐state electrolytes are preferentially utilized to ensure the integrity of the device (in this case electrolyte can also function as separator). Particularly, polymer or gel electrolytes can help to maintain the natural flexibility of electrodes. To date, the dominant flexible energy storage devices are highly focused on SCs and Li‐ion batteries. For instance, Chen et al. reported a stretchable, wire‐shaped SC using electrodes of carbon nanotube thin film continuously wrapped around prestretched elastic wires.^148^ CV curves for this SC remain almost unchanged when bent to different states. The flexibility of such a wire‐shaped device was further demonstrated by stretching from 0% to 370% while only losing slight capacitance beyond 250% stretch. Zheng co‐workers[[qv: 26b,d]] reported the large‐scale fabrication of flexible and wearable high‐performance SCs. RGO/Ni cotton composite based solid‐state SC yarns[[qv: 26c]] delivered maximum energy density and power density of 6.1 mWh cm^−3^ and 1400 mW cm^−3^, respectively.

Making BSH devices flexible would be an amazing way to realize advanced flexible energy storage systems. In particular, binder‐ and additive‐free 3D structured electrodes take more advantages to meet the increasing demands of high energy and power densities as well as good mechanical strain capability.^153^ Nevertheless, BSHs were extensively investigated for large‐scale applications such as hybrid electric vehicles, and little attention has been paid to flexible BSHs. In 2015, our group fabricated a flexible Li‐ion BSH by using 3D electrodes of multiwalled carbon nanotube network film and Li_4_Ti_5_O_12_ nanowire array, which were both grown directly on carbon cloth current collector (Figure 14b).^50^ This was the first report on flexible Li‐ion BSH device. With LiPF_6_‐based organic electrolyte, the device exhibited 3 V output voltage and impressive cycling stability up to 3000 cycles. Furthermore, based on the total volume of the full‐cell device, maximum energy, and power density of 4.38 mWh cm^−3^ and 560 mW cm^−3^ could be obtained, respectively. The energy density value was superior to those of previous thin‐film SCs fabricated directly on carbon cloth and even comparable to the commercial thin‐film lithium battery; the power density also approached that of the commercial 5.5 V/100 mF SC (can be charged within 3 s; Figure 14c). In another example, we developed a flexible alkaline CNTs//Fe_3_O_4_—C BSH device, in which the electrolyte was quasi‐solid‐state PVA‐KOH.^116^ This device exhibited three‐ to fourfold higher volumetric energy density than conventional iron oxide‐based flexible asymmetric SCs. In particular, it demonstrated high environmental suitability; good electrochemical attributes could still be maintained in cases of substantially bending, high mechanical pressure and elevated temperature (up to 80 °C), as shown in Figure 14d–f.

There are several other flexible BSHs were assembled and reported. For example, the CoO@PPy//activated carbon BSH device was investigated by Liu and co‐workers.^152^ In this work, well‐aligned CoO@PPy hybrid nanowire array positive electrode was grown directly on flexible nickel foam(Figure 14g); high electrochemical activities of both CoO and PPy were utilized to boost the electrochemical performance. An elegant synergistic interaction between cobalt oxide and PPy coating layer was discovered, which led to ≈3.3 times increase of the areal capacity. For the device, high energy density (≈43.5 Wh kg^−1^; ≈3 mWh cm^−3^) and outstanding cycleability (≈20 000 times) were demonstrated. Interestingly, two devices assembled in series could light light‐emitting diode(LED) indicators efficiently and drive a mini 130 rotation motor robustly (3 V, 0.45 W) (Figure 14h,i). With the BSH powering, the red LED was even effective enough for indication after 1 h. Xu et al.^154^ reported the design and synthesis of flexible Co_9_S_8_//Co_3_O_4_@RuO_2_ hybrid device which was cycled in the voltage range of 0–1.6 V and exhibited energy density of 1.21 mWh cm^−3^ at power density of 13.29 W cm^−3^. A CoMoO_4_@CoNiO_2_//AC flexible all‐solid‐state alkaline BSH had energy density of 59.75 Wh kg^−1^ at power density of 1464 W kg^−1^ with long stability of 50 000 cycles.^155^ Other Co‐ and V‐based solid‐state flexible BSH devices also exhibited high electrochemical performances, such as Co_3_O_4_//carbon^156^ (17.9 Wh kg^−1^ at 750 W kg^−1^) and VO*_x_*//VN^157^ (maximum energy and power densities are 0.61 mWh cm^−3^ and 0.85 W cm^−3^, respectively).

Transparency is an attractive feature for future optoelectronic devices such as screen displays, electrochromics,^158^ and photovoltaics.^159^ Besides flexibility, significant efforts are required to integrate BSHs with this functionality for powering such optoelectronic devices. Although transparent BSH devices have not yet been reported, there were increasing interests in developing transparent and flexible electrodes. Well‐known flexible transparent electrode materials contain carbon nanotube,^160^ graphene,^161^ conducting polymers,^162^ inorganic oxide coated polymers,^163^ metal nanowires,^164^, ^165^ and metal grids, etc.^166^ Among these, carbon nanomaterials especially graphene and carbon nanotubes are widely utilized owing to their outstanding electrical, optical, and mechanical properties.^167^, ^168^ Using carbon‐based electrodes, conventional electrochemical energy storage devices such as SCs have been made with flexibility and transparency, which would shed great light on some critical technologies for constructing transparent BSHs.

**Figure 15:**
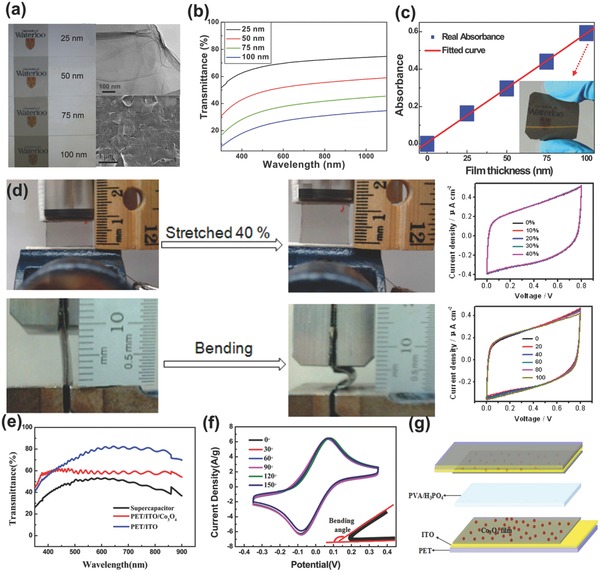
a) Digital photographs, b) transmittance spectra, and c) absorbance at 550 nm versus thickness of flexible graphene films. Reproduced with permission.^161^ Copyright 2010, American Institute of Physics. d) Digital photographs and CV curves of transparent and stretchable graphene SC before and after stretching and bending. Reproduced with permission.^169^ Copyright 2013, American Chemical Society. e) Transmittance spectra, f) bending testing, and g) schematic illustration of transparent and flexible Co_3_O_4_ electrode/SC. Reproduced with permission.^172^ Copyright 2016, The Royal Society of Chemistry.

Yu et al.^161^ prepared transparent graphene films with four different thicknesses (25, 50, 75, and 100 nm) (**Figure** **15**a) and examined their electrochemical behaviors. The transmittance spectra of the films are shown in Figure 15b, and the highest transmittance is ≈70% at 550 nm (25 nm thickness film). Absorbance increases linearly with film thickness (Figure 15c), and the transparency of graphene electrode could be tuned rationally from 0% to 97.7%. Dai and co‐workers^169^ fabricated a transparent and stretchable SC based on wrinkled graphene electrodes and polymer electrolyte, in which graphene and polymer electrolyte further functioned as current collector and separator, respectively. This transparent symmetric SC displayed excellent stretchability and flexibility. As shown in Figure 15d, their CVs and capacitances were almost unchanged even when the device was stretched up to 40% strain or bent to a large bending radius. By using carbon as electrode for transparent SCs, ultralong life time could be achieved. For instance, nanoengineered carbon films had 84% capacitance retention after 10 000 cycles,^170^ and CNF‐[RGO]*_n_*‐based SC maintained 81% of initial capacitance after 5000 cycles.^171^ But due to the intrinsic low capacitance of carbon materials and required low loading mass to achieve high transparency, faradaic charge storage processes are highly needed to boost the areal capacity of transparent electrodes and devices.

Very recently, Yang and co‐workers reported a flexible, transparent transition metal oxide nanocrystal electrode.^172^ As shown in Figure 15e, the transmittance of the electrode is ≈58%, indicating satisfied transparency. The CV curves retained the same after bending electrodes at different angles from 0° to 150° (Figure 15f), demonstrating fine flexibility of Co_3_O_4_ electrodes. But in this work, the assembled transparent electrochemical SC device was symmetric (based on the same Co_3_O_4_ film cathode and anode, Figure 15g); in this case, both cathodic and anodic processes could not entirely utilize the faradaic reaction of Co_3_O_4_, significantly deteriorating the full‐cell performance. Co_3_O_4_ is a typical battery‐type electrode material, in principle, it is better to be assembled with capacitive electrodes to form BSHs or to be paired with another battery‐type electrode to obtain batteries. Nevertheless, the high transparency, good flexibility, and superlong cycling life (93% retention after 30 000 CV cycles) still revealed the good prospect of advanced flexible and transparent faradaic oxide electrodes.

In future, the integration of BSHs with other functions such as electrochromism, shape memory, and even self‐healing, etc. will be very attractive and can be expected.

## 5 Conclusions and Outlook

In emerging fields such as electrification of transportation and smart miniaturized electronics, technologies require much larger amounts of energy to be stored within much shorter time but at low cost. Therefore, substantial increase of both the energy and power densities of energy storage systems is highly necessary. Replacing one capacitive electrode of a symmetric SC with a battery electrode allows the generation of an attractive BSH device that is with wider cell voltage and larger capacity (thus higher energy density). With appropriate battery‐electrode architecture design, BSHs would possibly have high power density approaching conventional SCs. In this review, we have addressed several existing and emerging kinds of BSH devices. Typical examples of these devices (cathode, anode, electrolyte, cycling performance, energy/power densities, voltage, etc.) have been summarized in **Table** **1**. With over twenty years of development, some technologies such as Li‐ion BSH have been successfully commercialized. Nevertheless, the overall performance of BSH devices (particularly the energy and power densities) is still not so competitive with advanced batteries and SCs. In addition, with the consideration of sustainable development of modern society, non‐Li‐ion BSHs with naturally abundant resources and multifunctionalities are indispensable; most kinds of BSHs are still at their early stage and need substantial advancement. Based on the above, several future trends and challenges are discussed here.

**Table 1:** Typical examples of reported BSH devices and performance

Device	Electrolyte	Cycle performance	Energy density	Power density	Voltage [V]
Li‐ion BSH					
CNTs//Li_4_Ti_5_O_12_ 50	Organic LiPF_6_	92%, 3000 cycles, 0.65 mA cm^−2^	4.38 mWh cm^−3^ at 13.5 mW cm^−3^		0.1–3.0
N‐Doped graphene//Li_4_Ti_5_O_12_ 51	Organic LiPF_6_	64%, 10 000 cycles, 1.5 A g^−1^	70 Wh kg^−1^ at 200 W kg^−1^; 21 Wh kg^−1^ at 8000 W kg^−1^		1–3
3DGraphene//Fe_3_O_4_/G^53^	Organic LiPF_6_	70%, 1000 cycles, 2 A g^−1^	147 Wh kg^−1^ at 150 W kg^−1^; 86 Wh kg^−1^ at 2587 W kg^−1^		1.0–4.0
AC//LiNi_0.5_Mn_1.5_O_4_ 57	Organic LiPF_6_	≈81%, 3000 cycles, 1 A g^−1^	≈19 Wh kg^−1^ at ≈150 W kg^−1^; ≈8 Wh kg^−1^ at ≈2.5 kW kg^−1^		1.5–3.25
LiNi_0.5_Mn_1.5_O_4_//AC^58^	Organic LiPF_6_	89%, 4000 cycles, 10 C	≈40 Wh kg^−1^ at ≈1 kW kg^−1^; 63 Wh kg^−1^ at ≈100 W kg^−1^		0–3.3
Li_3_V_2_(PO_4_)_3_–C//AC^59^	Organic LiPF_6_	66%, 1000 cycles	≈27 Wh kg^−1^ at 255 kW kg^−1^		0.5–2.75
SGCNT//UC‐LTO^65^	Organic LiBF_4_	–	40–45 Wh L^−1^ at 0.1–1 kW L^−1^; 28 Wh L^−1^ at 10 kW L^−1^		–
AC//prelithiated mesocarbon 62	Organic LiPF_6_	97%, 1000 cycles, 2C	92.3 Wh kg^−1^ maximum	5.5 kW kg^−1^ maximum	2.0–4.0
AC//Li_4_Ti_5_O_12_‐G^168^	Organic LiPF_6_	71%, 10 000 cycles, 10 C	50 Wh kg^−1^ at 10 W kg^−1^; 15 Wh kg^−1^ at 4000 W kg^−1^		≈1–2.5
AC//Hard carbon^173^	Organic LiPF_6_	83%, 10 000 cycles, 10 C	60 Wh kg^−1^ at ≈2350 W kg^−1^		1.5–3.9
NAC//Si/C^174^	Organic LiPF_6_	76.3%, 8000 cycles, 1.6 A g^−1^	237 Wh kg^−1^ at 867 W kg^−1^; 141 Wh kg^−1^ at 30127 W kg^−1^		2.0–4.5
LiTi_2_(PO_4_)_3_//AC^67^	Aqueous Li_2_SO_4_	85%, 1000 cycles, 10 mA cm^−2^	24 Wh kg^−1^ at 200 W kg^−1^; 15 Wh kg^−1^ at 1.0 kW kg^−1^		0–1.6
Na‐ion BSH					
Nanosheet carbon//ordered carbon^82^	Organic NaClO_4_	79%, 1000 cycles; 66%, 10 000 cycles, 6.4 A g^−1^	201 Wh kg^−1^ at 285 W kg^−1^; 50 Wh kg^−1^ at 16.5 kW kg^−1^		1.5–4.2
V_2_O_5_‐CNT//AC^83^	Organic NaClO_4_	80%, 900 cycles, 60 C	38 Wh kg^−1^ at 140 W kg^−1^; 7.5 Wh kg^−1^ at 5000 W kg^−1^		0–2.8
AC//Na‐titanate nanotubes^85^	Organic NaClO_4_	80%, 1000 cycles, 0.25 A g^−1^	34 Wh kg^−1^ at 100 W kg^−1^; ≈12.5 Wh kg^−1^ at 789 W kg^−1^		0.1–2
NaMnO_2_//AC^86^	Aqueous Na_2_SO_4_	97%, 10 000 cycles, 10C	13.2 Wh kg^−1^ at 1.0 kW kg^−1^		0–1.9
CoHCF//carbon microspheres^88^	Aqueous Na_2_SO_4_	92%, 1000 cycles, 2 A g^−1^	54.4 Wh kg^−1^ at 800 W kg^−1^; 37.8 Wh kg^−1^ at 5037 W kg^−1^		0–2
Na_4_Mn_9_O_18_//AC^89^	Aqueous Na_2_SO_4_	84%, 4000 cycles, 0.5 A g^−1^	34.8 Wh kg^−1^ at 62 W kg^−1^; 21.0 Wh kg^−1^ at 337.4 W kg^−1^		0–1.7
Acidic BSH					
PbO_2_//carbon^96^	0.1 m CH_3_SO_3_H/Pb(NO_3_)_2_+4 m NaNO_3_	100%, 5000 cycles, 22 C	29 Wh kg^−1^	–	0.7–1.7
PbO_2_//AC^98^	Aqueous H_2_SO_4_	83%, 3000 cycles, 4 C	26.5 Wh kg^−1^ at 30.8 W kg^−1^; 17.8 Wh kg^−1^ at 500 W kg^−1^		0.8–1.8
PbO_2_//AC^95^	Aqueous H_2_SO_4_	80%, 3000 cycles, 10 C	27 Wh kg^−1^ at 152 W kg^−1^; 18 Wh kg^−1^ at 691 W kg^−1^		0.65–1.88
Alkaline BSH					
AC//NiO—AC NFs^99^	Aqueous KOH	88%, 5000 cycles, 10 A g^−1^	43.75 Wh kg^−1^ at 7.5 kW kg^−1^		0–1.5
Ni(OH)_2_/graphene//graphene 101	Aqueous KOH	94.3%, 3000 cycles, 100 mV s^−1^	77.8 Wh kg^−1^ at 174.7 W kg^−1^; 13.5 Wh kg^−1^ at 15.2 kW kg^−1^		0–1.6
NiMoO_4_//AC^103^	Aqueous KOH	14.3%, 10 000 cycles, 5 A g^−1^	60.9 Wh kg^−1^ at 850 W kg^−1^; 41.1 Wh kg^−1^ at 17002 W kg^−1^		0–1.7
Ni_3_S_2_/MWCNT‐NC//AC^105^	Aqueous KOH	91%, 5000 cycles, 4 A g^−1^	19.8 Wh kg^−1^ at 798 W kg^−1^; 15.4 Wh kg^−1^ at 6.4 kW kg^−1^		0–1.6
(Cu,Ni)O NW//AC^106^	Aqueous KOH	100.9%, 6000 cycles, 5 mA cm^−2^	50.3 Wh kg^−1^ at 82.4 W kg^−1^		0–1.8
Co_3_O_4_@MWCNTs//AC^108^	Aqueous KOH	100%, 5500 cycles, 10 A g^−1^	31 Wh kg^−1^ at 3 kW kg^−1^		0–1.8
AC//Ni—Co oxide^109^	Aqueous KOH	85%, 2000 cycles, 8 mA	7.4 Wh kg^−1^ at 1902.9 W kg^−1^		0–1.2
CoMoO_4_‐3D graphene//AC^107^	Aqueous KOH	87.42%, 10 000 cycles, 1.67 A g^−1^	21.1 Wh kg^−1^ at 300 W kg^−1^; 3.59 Wh kg^−1^ at 6 kW kg^−1^		0–1.8
CNTs//Fe_3_O_4_—C^116^	Aqueous KOH KOH‐PVA Gel	67.6%, 1000 cycles, 5.5 mA cm^−2^	1.56 mWh cm^−3^ at 0.028 W cm^−3^		0–1.6
CoO@ppy//AC^152^	Aqueous NaOH	91.5%, 20 000 cycles, 25 mA cm^−2^	43.5 Wh kg^−1^ at 87.5 kW kg^−1^; 11.8 Wh kg^−1^ at 5.5 kW kg^−1^		0–1.8
BSH with redox electrolytes					
Pica//Pica 123	p‐BQ/PYR_14_TFSI (ionic liquid)	≈50%, 1000 cycles, 10 mA cm^−2^	27 Wh kg^−1^ at ≈420 W kg^−1^; ≈12 Wh kg^−1^ at ≈1 kW kg^−1^		0–3
AC//AC^124^	Cu(II)/[EMlm]BF_4_ (ionic liquid)	91%, 500 cycles	45 Wh kg^–1^	–	0–2
SWNT//SWNT^133^	DmFc/TBAP/THF (organic)	88.4%, 10 000 cycles, 5 A g^−1^	36.76 Wh kg^–1^ at 1.04 kW kg^–1^	–	0–2.1
AC//AC^134^	HQ/H_2_SO_4_ (aqueous)	65%, 4000 cycles, 4.42 mA cm^−2^	31.3 Wh kg^−1^	–	0–1
BSH with psuedocapacitive electrodes					
Na_2_Fe_2_(SO_4_)_3_//Ti_2_CT*_x_* 140	Organic NaPF_6_	96%, 100 cycles, 600 mA g^−1^	260 Wh kg^−1^ at 1.4 kW kg^−1^ (based on the weight of Ti_2_CT*_x_*)		0.1–3.8
MnO_2_//Bi_2_O_3_ 141	Aqueous Na_2_SO_4_	85%, 4000 cycles, 6 mA cm^−2^	11.3 Wh kg^−1^ at 352.6 W kg^−1^; 9.1 Wh kg^−1^ at 3370 W kg^−1^		0–1.8

First of all, searching for new materials is still highly essential to develop advanced BSH devices, especially low‐cost Na‐ion BSH as well as BSH with redox electrolytes, etc. Electrode and electrolyte materials are two key factors determining the device's performance. In general, the redox potential, crystal structure, electrical conductivity, capacity, and chemical/electrochemical stability should be considered when choosing the electrode materials; while for electrolyte, its potential window, ionic conductivity, thermal stability, chemical inertness, and compatibility with electrodes are the first to be taken into account.

➢ Specifically, for Na‐ion BSH, the most urgent task is to manufacture high‐capacity Na‐ion electrodes with fast electrochemical kinetics; with capacity comparable to Li‐ion electrodes, Na‐ion BSH might achieve energy storage capability at the same level to Li‐ion BSH.
➢ In BSH with redox electrolytes, finding new multielectron redox‐active species that can be incorporated into electrolytes is important for advancing the device's performance.

To accomplish these goals, not only extensive experiment exploring but also computational studies relating to theory, modeling, and simulation are highly desirable. Computational research can provide oriented guidance for selecting electrode materials, electrolytes, and even for designing high‐quality interfaces and ideal architectures.^20^

Another direction is further engineering the micro‐/nanostructures of typical electrode materials of BSHs. This aims to boosting the redox kinetics of battery‐type electrode and enhancing the capacitance of the capacitive electrode, thus eventually gaining both high energy and power densities of the device.

➢ In principle, sluggish redox processes occur in battery‐type electrode and thus hinder the power performance. Nanostructuring that reduces the ion diffusion pathway and hybridization at the nanoscale with target species (such as carbon/conducting polymers)[[qv: 6c,7b,53]] that increases the electrical conductivity and stability are two state‐of‐the‐art strategies to address this issue. Besides nanoscale engineering, the future research will focus on doping or surface/interface design to pursue the goals of fast electron transport and rapid ion/mass diffusion.
➢ For capacitive electrode, challenge still remains in increasing the capacitance, which is critical for charge/weight/volume balance with the battery‐type electrode to maximize the energy density of the device.^168^, ^173^, ^174^, ^175^, ^176^ However, two possible solutions are available: (i) construction of highly mesoporous conductive carbon that possesses increased active surface to accumulate ions or heteroatom‐functionalized carbon that can suffer from surface redox reaction, contributing pseudocapacitance; (ii) replacing carbon materials with advanced pseudocapacitive materials, especially high‐capacitance “intercalation pseudocapacitive oxides” such as MoO_3_ and Nb_2_O_5_, and highly conductive 2D MXenes, etc.
➢ To enable the above strategies to work efficiently, it is essential to design 3D hybrid electrode architectures, which have been widely implemented in batteries and SCs.^64^, ^153^ Within such structure, 3D continuous electrical conduction pathway and 3D mesoporosity exist, enabling rapid electron and ion accessibility to electrochemically active sites and minimizing the electrode strain during charging/discharging.

**Figure 16:**
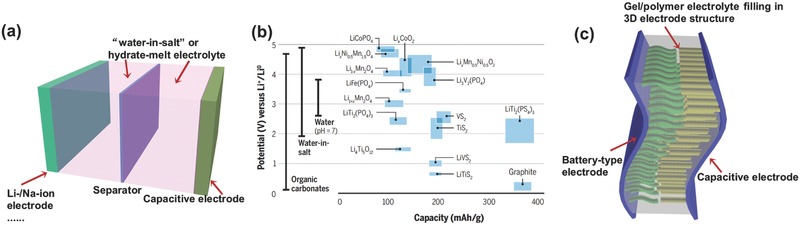
a) Conceptual illustration of future BSHs based on “water‐in‐salt” or hydrate‐melt electrolytes. b) The Li storage materials system that can be utilized with “water‐in‐salt” electrolyte. The comparison with the cases in simple aqueous electrolyte and organic carbonates electrolyte is also presented. Reproduced with permission.^172^ Copyright 2015, Science, AAAS. c) Schematic illustration of a conceptual flexible BSH using 3D nanostructure arrays as the electrode integrated with gel or polymer electrolyte.

Third, aqueous BSH holds great advantages of low cost, high safety, and high power; it is a green and sustainable electrochemical energy storage system. Nevertheless, even with its asymmetric device configuration, the theoretical operating voltage was limited to 2 V due to the electrolysis of water, precluding the use of high‐voltage electrode couples that enable the high energy density. Further widening of the voltage window is always very challenging.^177^ Recent breakthrough in aqueous Li‐ion batteries has shed great light on aqueous BSHs; exciting “water‐in‐salt” electrolytes^178^ and hydrate‐melt electrolytes^179^ were suggested to overcome the water electrolysis limitation, which can push the battery's voltage window up to 3–4 V. Based on this progress, a concept for future aqueous BSHs is schematically depicted in **Figure** **16**a. In such a device design, “water‐in‐salt” electrolyte or hydrate‐melt electrolyte are utilized, and the battery‐type electrode can either be Li‐/Na‐ion battery cathode or anode that are typically utilized in batteries with organic electrolytes. Figure 16b displays the possible Li‐ion battery electrode materials that can be considered with “water‐in‐salt” electrolytes.^180^ It is expected that the great increase in device voltage would enrich the electrode materials system of aqueous BSHs and substantially lead to high energy density.

Fourth, as BSH devices can simultaneously have high energy and power densities, it is clear that they are highly desirable to power future portable/smart/wireless electronics or optoelectronics. To this end, multifunctional BSHs which may be flexible, bendable, stretchable, and even transparent as well as with sensing functions to ambient environment need in‐depth study. To date, only quite limited flexible Li‐ion and alkaline BSH devices have been reported and their performance has not yet optimized. With similar device architecture and electrochemistry, other kinds of flexible BSH devices can be anticipated; relating technologies can be directly learnt from flexible Li‐ion or alkaline BSH devices as well as flexible SCs and batteries. Future development of multifunctional BSHs is likely to start with flexible Na‐ion BSH and gradually goes to other types of flexible BSHs, and further goes well beyond. In particular, to accomplish the goal of storing more energy quickly in BSH, the above mentioned 3D electrode structure will be very helpful, especially 3D nanostructure arrays. This structure can adapt to the shape deformation of current collector and increase the mass loading as compared to conventional nanostructured thin‐film electrodes (without deteriorating the ion diffusion or even transparency). An ideal prospect is further introduction of gel or polymer electrolytes into the interspacing of 3D structure, which enables intimate interfacial contact between active material and electrolyte (Figure 16c). The use of gel/polymer electrolyte eliminates the need for a separator, reduces the open spaces/volume and enhances the mechanical properties of the device. This design is believed to push forward the development of future multifunctional BSHs.
